# Temperature-tunable Fano resonance induced by strong coupling between Weyl fermions and phonons in TaAs

**DOI:** 10.1038/ncomms14933

**Published:** 2017-03-30

**Authors:** B. Xu, Y. M. Dai, L. X. Zhao, K. Wang, R. Yang, W. Zhang, J. Y. Liu, H. Xiao, G. F. Chen, S. A. Trugman, J-X Zhu, A. J. Taylor, D. A. Yarotski, R. P. Prasankumar, X. G. Qiu

**Affiliations:** 1 Beijing National Laboratory for Condensed Matter Physics, Institute of Physics, Chinese Academy of Sciences, P.O. Box 603, Beijing 100190, China; 2 Center for High Pressure Science and Technology Advanced Research, Beijing 100094, China; 3 Center for Integrated Nanotechnologies, Los Alamos National Laboratory, Los Alamos, New Mexico 87545, USA; 4 Collaborative Innovation Center of Quantum Matter, Beijing 100190, China; 5 Theoretical Division, Los Alamos National Laboratory, Los Alamos, New Mexico 87545, USA; 6 Associate Directorate for Chemistry, Life and Earth Sciences, Los Alamos National Laboratory, Los Alamos, New Mexico 87545, USA

## Abstract

Strong coupling between discrete phonon and continuous electron–hole pair excitations can induce a pronounced asymmetry in the phonon line shape, known as the Fano resonance. This effect has been observed in various systems. Here we reveal explicit evidence for strong coupling between an infrared-active phonon and electronic transitions near the Weyl points through the observation of a Fano resonance in the Weyl semimetal TaAs. The resulting asymmetry in the phonon line shape, conspicuous at low temperatures, diminishes continuously with increasing temperature. This behaviour originates from the suppression of electronic transitions near the Weyl points due to the decreasing occupation of electronic states below the Fermi level (*E*_F_) with increasing temperature, as well as Pauli blocking caused by thermally excited electrons above *E*_F_. Our findings not only elucidate the mechanism governing the tunable Fano resonance but also open a route for exploring exotic physical phenomena through phonon properties in Weyl semimetals.

## Abstract (web summary)

The study of lattice vibrations coupled to electronic excitations may provide an avenue for exploring exotic physical phenomena. Here, Xu *et al*. observe a Fano resonance in the Weyl semimetal TaAs, revealing evidence for a strong coupling between phonons and Weyl fermions.

The Weyl semimetal (WSM) phase, a novel topological state of quantum matter, has been proposed to exist in materials with two non-degenerate bands crossing at *E*_F_ in three-dimensional momentum space^1^. At the band crossing points (Weyl points), the electronic dispersion is linear in all three directions, resembling a three-dimensional version of graphene, and the low-energy excitations can be described by Weyl equations, producing a condensed-matter realization of Weyl fermions^2^. Recently, such a WSM state has been discovered in non-centrosymmetric transition-metal monoarsenides and monophosphides (TaAs, TaP, NbAs and NbP)^3^,^4^,^5^,^6^,^7^,^8^, where 12 pairs of Weyl points have been found. Since the Weyl points are located in close proximity to *E*_F_ in these materials, interband electronic transitions near the Weyl points occur at a very low energy, 2|*μ*|, where |*μ*| represents the chemical potential with respect to the Weyl points^9^,^10^. This energy scale overlaps optical phonon frequencies^11^. Consequently, strong coupling between electronic transitions near the Weyl points (Weyl fermions) and phonons may arise, manifested through a temperature-tunable Fano resonance.

This is actually a rare occurrence. Fano resonances generally do not occur in conventional metals or semiconductors, since the energy scale of interband electronic transitions in these materials is usually much higher than phonon excitations. However, this interesting phenomenon has been reported in bilayer or few-layer graphene^12^,^13^,^14^, topological insulators^15^,^16^ and some strongly correlated electron systems, such as stripe-phase nickelates^17^ and high-*T*_c_ superconductors^18^,^19^. The observed Fano resonance in strongly correlated materials is very weak, with 1/*q*^2^, a parameter that describes the asymmetry of the Fano line shape, only reaching ∼0.04 (refs 17, 18, 19). In topological insulators, the effect is also weak 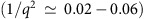 and cannot be observed without manipulating extrinsic parameters, such as magnetic field^15^, chemical doping^16^ or micro-fabrication on the surface^20^. In contrast, bilayer or few-layer graphene exhibits a strong Fano resonance, with 1/*q*^2^≥≥1, but the resonance energy is ∼1,600 cm^−1^ (200 meV), much greater than *E*_F_, making it impossible to tune the resonance via temperature^12^,^13^,^14^. Moreover, the Fano physics revealed in all the above materials is associated with or has considerable contributions from coupling between phonons and conventional massive fermions.

Here we observe an intrinsic, strong 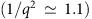 and temperature-tunable Fano resonance, which arises purely from quantum interference between phonons and massless Weyl fermions, in the recently discovered Weyl semimetal TaAs. We further demonstrate that the Fano line shape can be tuned by changing the occupation of the electronic states near the Weyl points. These observations not only open a novel avenue for exploring exotic quantum phenomena in WSMs, such as the chiral anomaly^21^,^22^,^23^,^24^, but also set the stage for a variety of potential applications that take advantage of the ability to tune the Fano resonance using different parameters (for example, temperature, light or magnetic/electric fields).

## Results

### Sample growth and characterization

High-quality single crystals of TaAs were synthesized through a chemical vapour transport method^23^. The as-grown crystals are polyhedrons with shiny facets up to 1.5 mm in size. X-ray diffraction measurements reveal that the as-grown facets are the (001), (107) and (112) surfaces (Supplementary Fig. 1). Systematic optical measurements were carried out on all three surfaces.

### Reflectivity and optical conductivity

**Figure 1:**
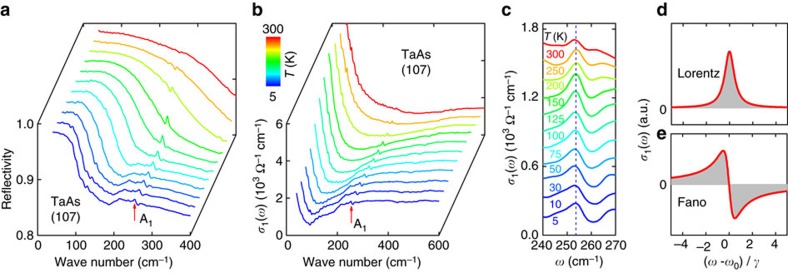
Reflectivity and optical conductivity of TaAs. (**a**) Reflectivity of TaAs in the far-infrared region measured at different temperatures on the (107) surface. (**b**) Optical conductivity of TaAs on the (107) surface up to 600 cm^−1^ at different temperatures. (**c**) Enlarged view of the optical conductivity in the region of the infrared-active A_1_ mode at ∼253 cm^−1^. (**d**) Schematic of the symmetric Lorentz oscillator, which describes the phonon line shape in the optical conductivity without strong electron–phonon coupling. (**e**) Schematic of the asymmetric Fano resonance, used to describe the phonon line shape in the presence of strong electron–phonon coupling.

Figure 1a shows the far-infrared reflectivity *R*(*ω*) measured on the (107) surface of TaAs at 11 different temperatures from 5 to 300 K (ref. 10). The relatively high *R*(*ω*) that approaches unity at zero frequency is consistent with the metallic nature of TaAs. A well-defined plasma edge in the far-infrared region suggests very low carrier density, in agreement with the tiny volumes enclosed by the Fermi surfaces in this material^3^,^4^,^5^,^6^. In addition to the broad features in *R*(*ω*), a sharp feature can be clearly identified at ∼253 cm^−1^ (31 meV), as indicated by the arrow, which is associated with the infrared-active A_1_ phonon mode (Supplementary Note 1).

In order to gain direct information about this mode, we calculated the optical conductivity *σ*_1_(*ω*) from *R*(*ω*) using a Kramers–Kronig analysis^10^ (more details in Methods section). Figure 1b displays *σ*_1_(*ω*) of TaAs on the (107) surface in the far-infrared region at different temperatures. The low-frequency *σ*_1_(*ω*) is dominated by a narrow Drude response alongside prominent linear features, whose origin and temperature dependence have been previously discussed in detail^10^. The A_1_ mode manifests itself as a sharp peak in the *σ*_1_(*ω*) spectrum, as indicated by the arrow. Figure 1c shows an enlarged view of *σ*_1_(*ω*) in the frequency region of 240–270 cm^−1^, where the A_1_ mode can be seen more clearly. It is well known that, in the absence of strong electron–phonon coupling, the phonon exhibits a symmetric line shape in *σ*_1_(*ω*) that can be described by a Lorentz oscillator, as schematically illustrated in Fig. 1d. In contrast, strong electron–phonon coupling gives rise to an asymmetric phonon profile in *σ*_1_(*ω*), known as the Fano line shape^13^,^14^,^25^ (Fig. 1e). As shown in Fig. 1c, the A_1_ mode in TaAs exhibits a striking asymmetric line shape at low temperatures, which is an unequivocal signature of strong electron–phonon coupling. More interestingly, the asymmetry of the phonon line shape diminishes as the temperature rises, suggesting that the coupling-induced Fano resonance in TaAs can be tuned by temperature.

### Mechanism governing the temperature-tunable Fano resonance

**Figure 2:**
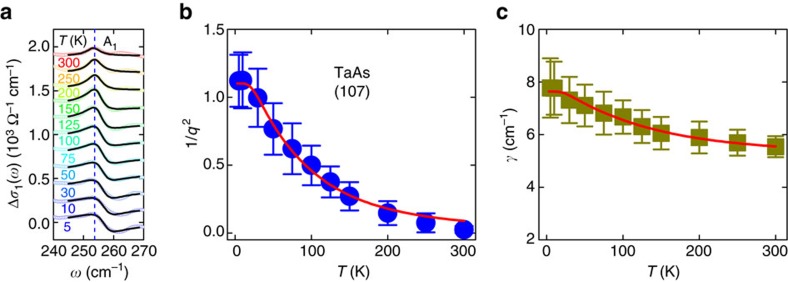
Fano fit and temperature dependence of fitting parameters. (**a**) Line shape of the A_1_ phonon, with the electronic background subtracted at different temperatures. The black solid lines through the data denote the Fano fitting results. (**b**,**c**) Temperature dependence of the Fano parameter 1/*q*^2^ and the line width *γ* of the A_1_ mode, respectively. Error bars for both parameters are estimated by fitting the phonon line shape to the Fano equation in different frequency ranges at all measured temperatures. The red solid lines through the data in each panel represent the modelling results.

To quantify the temperature dependence of the A_1_ mode, we extract the phonon line shape by subtracting a linear electronic background in a narrow frequency range at all measured temperatures, as shown in Fig. 2a. At each temperature, the phonon is fit with the Fano line shape^25^,


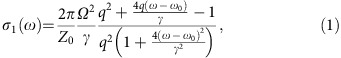


where *Z*_0_ is the vacuum impedance; *ω*_0_, *γ* and *Ω* correspond to the resonance frequency, linewidth and strength of the phonon, respectively; *q* is a dimensionless parameter that describes the asymmetry of the Fano profile. A larger 1/*q*^2^ indicates more conspicuous asymmetry in the phonon line shape, while for 1/*q*^2^=0, the symmetric Lorentz line shape is fully recovered. The solid lines in Fig. 2a represent the fitting curves, which describe the measured phonon line shapes reasonably well at all temperatures. This procedure also returns the temperature dependence of the fitting parameters.

Figure 2b depicts 1/*q*^2^ as a function of temperature. While 1/*q*^2^ adopts a large value of 1.1 at 5 K (refs 12, 13, 14, 18), it decreases dramatically with increasing temperature. This suggests that the A_1_ mode is strongly coupled to a continuum of electron–hole excitations, and the resulting Fano resonance varies significantly with temperature. We first trace the origin of this coupling by examining the band structure of TaAs. Both first-principle calculations and angle-resolved photoemission spectroscopic measurements have revealed 12 pairs of Weyl points in TaAs^3^,^4^,^5^,^6^. These Weyl points are categorized into two types^3^. Four pairs in the *k*_*z*_=0 plane, about 2 meV above *E*_F_, are defined as W1, while another eight pairs off the *k*_*z*_=0 plane, lying about 21 meV below *E*_F_, are named W2. Interband electronic transitions in the vicinity of a Weyl point start at *ω*=2|*μ*| (refs 9, 10), making it easy to calculate that electronic transitions near W2 turn on at *ω*=42 meV (∼336 cm^−1^). Thus electronic transitions at the frequency of the A_1_ mode (253 cm^−1^) do not occur near W2. This implies that the A_1_ mode is unlikely to be coupled to the electronic transitions near W2. However, electronic transitions near W1 set in at *ω*>4 meV (∼32 cm^−1^) and can therefore overlap with the frequency of the A_1_ mode, suggesting that this mode is coupled to the electronic transitions near W1.

**Figure 3:**
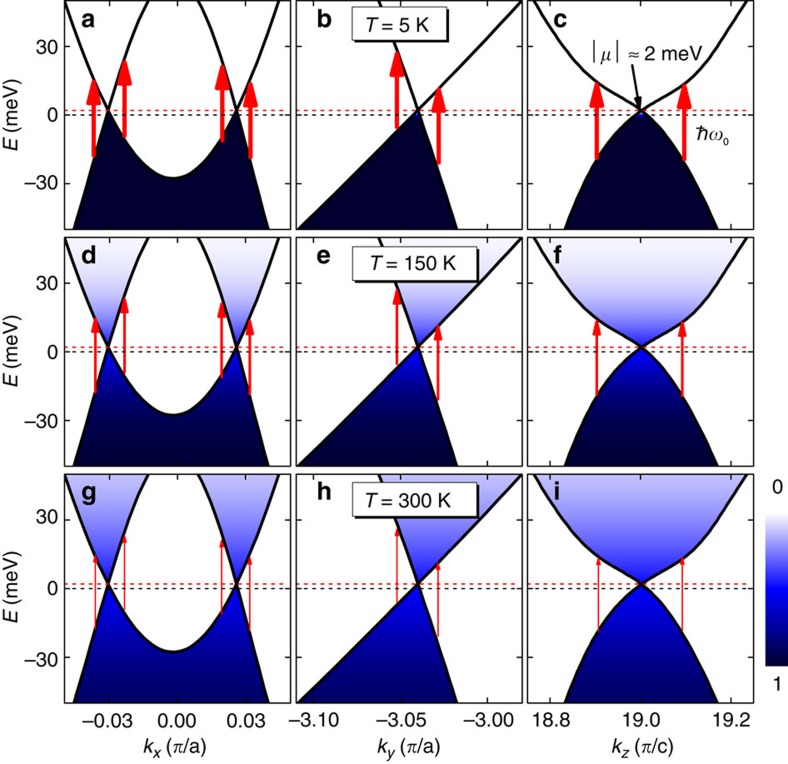
Occupation probability of the electronic states near W1. Band structure along three different momentum directions near the Weyl points W1 in TaAs. The black and red dashed lines in each panel correspond to the Fermi level and the energy of W1 (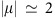 meV), respectively. The colour maps, which are calculated from the Fermi–Dirac distribution function *f*(*E*), denote the occupation probability of the electronic states at different temperatures of 5 K (**a**–**c**), 150 K (**d**–**f**) and 300 K (**g**–**i**). The red arrows represent the electronic transitions at the energy of the A_1_ mode *ħω*_0_. The thickness of each arrow schematically depicts the transition amplitude.

Having attributed the asymmetric line shape of the A_1_ mode to its strong coupling with the electronic transitions near W1, we proceed to understand the temperature dependence of this mode. In WSMs, since the Weyl points are in close proximity to *E*_F_, electronic transitions near the Weyl points can be dramatically affected by thermal excitations, thus changing the line shape of the phonon that is coupled to these transitions. Figure 3 shows the band structure along three momentum directions near W1 in TaAs. The occupation probabilities of the electronic states in these bands (colour maps) are calculated using the Fermi–Dirac distribution function at three different temperatures (5, 150 and 300 K), from which we see that the occupation of the electronic states near W1 depends strongly on the temperature. At 5 K (Fig. 3a–c), the electronic states below *E*_F_ are fully occupied (blue), while the states above *E*_F_ are empty (white). In this case, interband transitions at the energy of the A_1_ mode *ħω*_0_ are strong, as indicated by the thick arrows. As the temperature increases from 5 to 300 K, thermal excitations cause vacant states to appear below *E*_F_ and electronic states above *E*_F_ to be partially occupied. Electronic transitions at *ħω*_0_ are significantly suppressed (illustrated by the thin arrows in Fig. 3), because the available initial states for these transitions decrease, and many of the final states are Pauli blocked by thermally excited electrons. This suppression of the electronic transitions near W1 is directly responsible for the change in the A_1_ phonon line shape.

For a more quantitative analysis, the dimensionless parameter *q* in the Fano theory is given by^13^,^25^


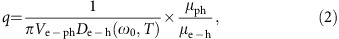


where *V*_e−ph_ is the electron–phonon coupling strength; *D*_e−h_(*ω*_0_, *T*) is the joint electron–hole pair density of states at the frequency of the A_1_ mode *ω*_0_ for a given temperature *T*; and *μ*_ph_ and *μ*_e−h_ represent the optical matrix elements for phonon and electron–hole pair excitations, respectively. In this equation, we note that raising *T* mainly modifies *D*_e−h_(*ω*_0_, *T*) by thermally exciting electrons to the electronic states above *E*_F_ and creating holes below *E*_F_. Near the Weyl points, the finite-temperature joint electron–hole pair density of states at *ħω*_0_ takes the form


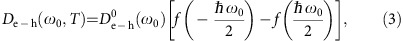


where 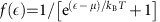 is the Fermi function, with 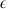 representing the energy of the single-particle state with respect to the Weyl points and 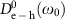 being the zero-temperature joint electron–hole pair density of states at *ħω*_0_. The red solid curve in Fig. 2b is the least-squares fit to the experimental temperature dependence of 1/*q*^2^ (blue solid circles) using equation (2). The excellent agreement between our experimental data and the model further underlines the intimate link between the line shape of the A_1_ mode and the electronic transitions near W1, which are continuously suppressed with increasing temperature due to the reduced occupation of the electronic states below *E*_F_ and Pauli blocking from thermally excited electrons above *E*_F_.

A careful examination of the temperature dependence of the A_1_ phonon linewidth *γ* (Fig. 2c) leads us to the same conclusion. In the case of weak electron–phonon coupling, phonon decay is dominated by the anharmonic effect: a zone-centre phonon decays into two acoustic modes with the same frequencies and opposite momenta^26^,^27^. The temperature dependence of the phonon linewidth *γ*^ph−ph^(*T*) for this process follows


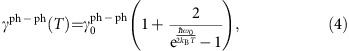


where *γ*_0_^ph−ph^ is the residual linewidth at zero temperature. Apparently, this model does not account for the behaviour of the A_1_ mode in TaAs, since it gives an increasing *γ* as the temperature is raised, which is opposite to our experimental observation. Instead, strong electron–phonon coupling must be taken into account to understand the temperature dependence of *γ* in TaAs. In a system with strong electron–phonon coupling, a phonon can also decay by creating an electron–hole pair^28^. This process is sensitive to *D*_e−h_(*ω*_0_, *T*) and is thus suppressed with increasing temperature due to thermal excitations, resulting in a temperature-dependent phonon linewidth *γ*^e−ph^(*T*)


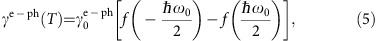


where *γ*_0_^e−ph^ represents a residual linewidth. Consequently, the temperature-dependent linewidth of a phonon mode that is strongly coupled to electronic excitations is given by *γ*(*T*)=*γ*^ph−ph^(*T*)+*γ*^e−ph^(*T*). This equation gives an excellent description to the measured temperature dependence of the linewidth for the A_1_ mode in TaAs, as shown by the red solid curve in Fig. 2c. The above observations explicitly demonstrate that the A_1_ mode is strongly coupled to the electronic transitions near W1, and both the line shape and linewidth of this phonon are closely tied to these transitions, which can be continuously tuned by temperature through varying the occupation of the electronic states near W1.

To lend further credence to our experimental results and analysis, we performed optical measurements at 12 different temperatures on the (112) surface. Essentially identical behaviour was revealed for the A_1_ mode (Supplementary Fig. 3). We then utilized the same methods and models to analyse the line shape and linewidth of this mode observed on the (112) surface (Supplementary Fig. 4), reaching the same conclusions.

Finally, we note that although electronic transitions at *ħω*_0_ are absent near W2 at low temperatures, Fermi smearing at high temperatures may relax the Pauli blocking, allowing these transitions to occur. From equations (2 and 3), we can easily calculate the asymmetry of the A_1_ mode arising from coupling to W2 at 300 K: 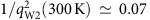. This small value of 1/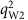, which vanishes quickly with decreasing temperature, suggests that electronic transitions near W2 do not give rise to a noticeable Fano resonance in the A_1_ mode of TaAs over the measured temperature range (5–300 K). However, the enhancement of these transitions with increasing temperature may produce a strong Fano resonance at high enough temperatures. It is also worth pointing out that changing the occupation of the electronic states near W1 via other methods, such as electrical gating or femtosecond optical excitation, should induce a similar change in the Fano line shape of the A_1_ mode in TaAs.

## Discussion

The strong coupling between Weyl fermions and phonons enables the study of exotic quantum phenomena in WSMs by tracking the properties of phonons. One interesting proposal derived from our findings is to provide experimental evidence for the chiral anomaly^21^,^22^,^23^,^24^, in which the application of parallel electric (**E**) and magnetic (**B**) fields pumps electrons from one Weyl point to the other with opposite chirality at a rate proportional to **E**·**B**, leading to a shift of *E*_F_ in opposite directions at different Weyl points. This *E*_F_ shift caused by the chiral anomaly can significantly affect the electronic transitions near the Weyl points, which accordingly changes the line shape of the phonon that is coupled to these transitions.

For more insight, we can calculate the chiral anomaly-induced *E*_F_ shift, given by^9^,^29^


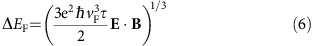


where *v*_F_ is the Fermi velocity and *τ* is the scattering time between different Weyl points. To obtain a realistic estimate, we take *v*_F_=5 × 10^5^ m s^−1^ (refs 6, 23, 30), *τ*=5 × 10^−11^ s (refs 9, 24, 29), **B**=5 T and **E**=1 V mm^−1^, giving Δ*E*_F_=31.34 meV. Such an *E*_F_ shift would completely block the electronic transitions at *ħω*_0_ near W1 (Fig. 3), resulting in a full recovery of the symmetric Lorentz line shape. This theoretical estimate thus suggests that, at low temperatures, the striking asymmetry of the A_1_ phonon line shape in TaAs should decrease with increasing **E**·**B**, due to the chiral anomaly-induced *E*_F_ shift, until a symmetric Lorentz line shape is fully recovered at high **E**·**B** fields. Experimental observation of this effect would be a strong evidence for the chiral anomaly in WSMs.

Furthermore, our observations suggest a variety of potential device applications. The Fano resonance in optical spectra arises from strong quantum interference between topologically non-trivial Weyl fermions and phonons, and the properties of Weyl fermions can be manipulated by a variety of different parameters, such as temperature, light^31^,^32^ and electric/magnetic fields^23^,^24^. This could thus lead to a new class of devices operating at the Fano resonance that could be controlled by any one of these parameters, making them unusually flexible and thus potentially useful in a wide range of applications.

In conclusion, we have found compelling evidence for strong coupling between the infrared-active A_1_ phonon mode and electronic transitions near W1 (Weyl fermions) in TaAs through the observation of a Fano resonance. Varying the temperature, which changes the occupation of the electronic states in proximity to *E*_F_, can continuously tune the amplitude of the electronic transitions near W1, thus manipulating the line shape of the A_1_ mode. Our findings not only suggest a new approach for investigating exotic quantum phenomena in WSMs, such as the chiral anomaly, through the behaviour of phonons, but also imply a broad range of potential applications, such as in thermo-, magneto- and electro-optical devices. Please note that, after our manuscript was submitted, we noticed two preprints (refs 33, 34) that theoretically propose detecting the chiral anomaly in WSMs through phonon dynamics.

## Methods

### Sample synthesis

High-quality TaAs single crystals were grown through a chemical vapour transport method^23^. A previously reacted polycrystalline TaAs was filled in a quartz ampoule using iodine (2 mg cm^−3^) as the transporting agent. After evacuating and sealing, the ampoule was kept at the growth temperature for 3 weeks. Large polyhedral crystals with dimensions up to 1.5 mm are obtained in a temperature field of Δ*T*=1,150–1,000 °C.

### Optical measurements

Near normal incident reflectivity R(*ω*) was measured over a broad frequency range on a Bruker VERTEX 80v FTIR spectrometer. In order to accurately measure the absolute *R*(*ω*) of the samples, an *in situ* gold overcoating technique^35^ was used. R(*ω*) from 40 to 15,000 cm^−1^ were measured at 11 different temperatures from 300 down to 5 K on three different surfaces of as-grown single crystals in an ARS-Helitran cryostat. Since a broad spectral range is required for a Kramers-Kronig analysis, we extended our data to the ultraviolet range (up to 50,000 cm^−1^) at room temperature using an AvaSpec-2048 × 14 fiber optic spectrometer.

### Kramers–Kronig analysis

The real part of the optical conductivity *σ*_1_(*ω*) has been determined via a Kramers–Kronig analysis of *R*(*ω*)^36^. Below the lowest measured frequency, we used a Hagen–Rubens (*R*=1−*A*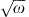) form for the low-frequency extrapolation. Above the highest measured frequency, we assumed a constant reflectivity up to 12.5 eV, followed by a free-electron (*ω*^−4^) response.

### Data availability

All data that support the findings of this study are available from the corresponding authors upon request.

## Additional information

**How to cite this article:** Xu, B. *et al*. Temperature-tunable Fano resonance induced by strong coupling between Weyl fermions and phonons in TaAs. *Nat. Commun.* **8,** 14933 doi: 10.1038/ncomms14933 (2017).

**Publisher's note**: Springer Nature remains neutral with regard to jurisdictional claims in published maps and institutional affiliations.

## Supplementary Material

Supplementary Information Supplementary Note, Supplementary Figures and Supplementary References
