# Supplementary material for: Temperature-tunable Fano resonance induced by strong coupling between Weyl fermions and phonons in TaAs
Source: Nat Commun. 2017 Mar 30;8:14933. doi: 10.1038/ncomms14933 (PMC5379101; doi:10.1038/ncomms14933)
Supplement: Supplementary Information — Supplementary Note, Supplementary Figures and Supplementary References [file ncomms14933-s1.pdf]

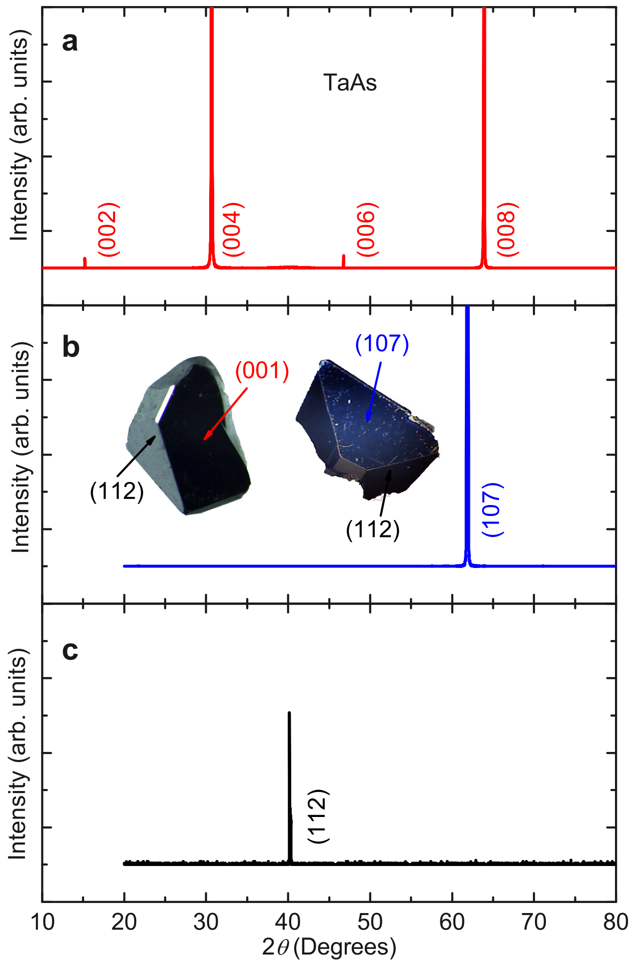

Supplementary Figure 1 | X-ray diffraction of TaAs. a–c, X-ray diffraction patterns from the (001), (107) and (112) surfaces of as-grown TaAs single crystals, respectively. The insets of **b** show the optical images of the measured single crystals, where the Miller indices are marked on corresponding surfaces.

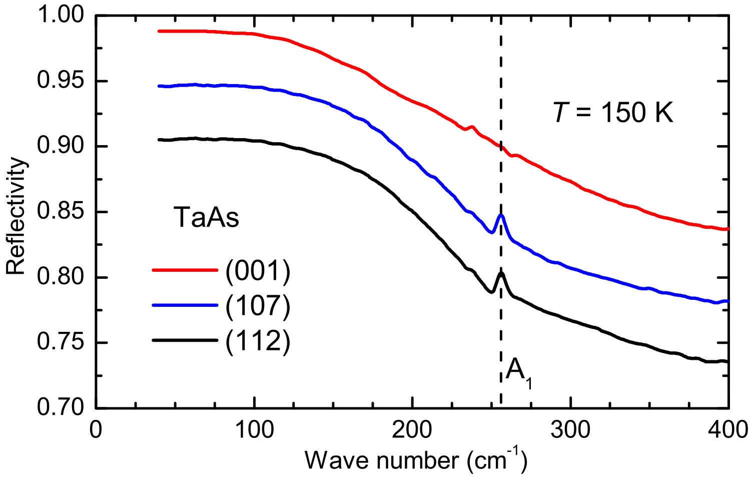

Supplementary Figure 2 | Far-infrared reflectivity of TaAs. Reflectivity $R(\omega)$ in the far-infrared region measured at 150 K on three different surfaces of TaAs: (001) (red curve), (107) (blue curve), and (112) (black curve). The sharp peak at $\sim$253 cm^{-1} in $R(\omega)$ measured on the (107) and (112) surfaces is associated with the IR-active A_1 mode. This mode is active along the c axis, in good agreement with the fact that it is absent in the $R(\omega)$ spectrum measured on the (001) surface.

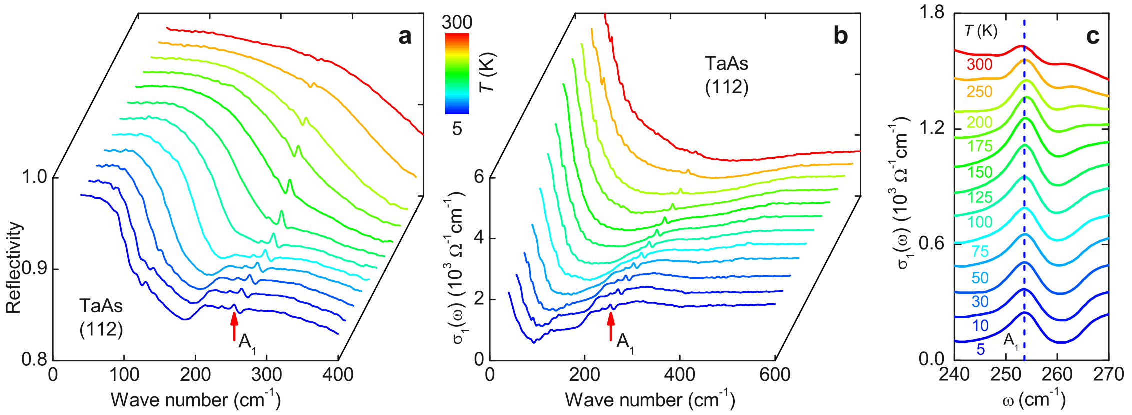

Supplementary Figure 3 | Reflectivity and optical conductivity of TaAs. **a**, Far-infrared $R(\omega)$ of TaAs measured on the (112) surface at 12 different temperatures from 5 to 300 K. **b**, Optical conductivity $\sigma_1(\omega)$ of TaAs on the (112) surface at different temperatures calculated from $R(\omega)$ using a Kramers-Kronig analysis. The sharp feature associated with the A_1 mode is indicated by a red arrow in both the $R(\omega)$ and $\sigma_1(\omega)$ spectra. **c**, Enlarged view of the optical conductivity in the frequency range of the IR-active A_1 mode. The line shape of this mode exhibits pronounced asymmetry at low temperatures, signaling strong electron-phonon coupling. The asymmetry of the phonon line shape varies dramatically with temperature. All of these observations are identical to the ones made on the (107) surface.

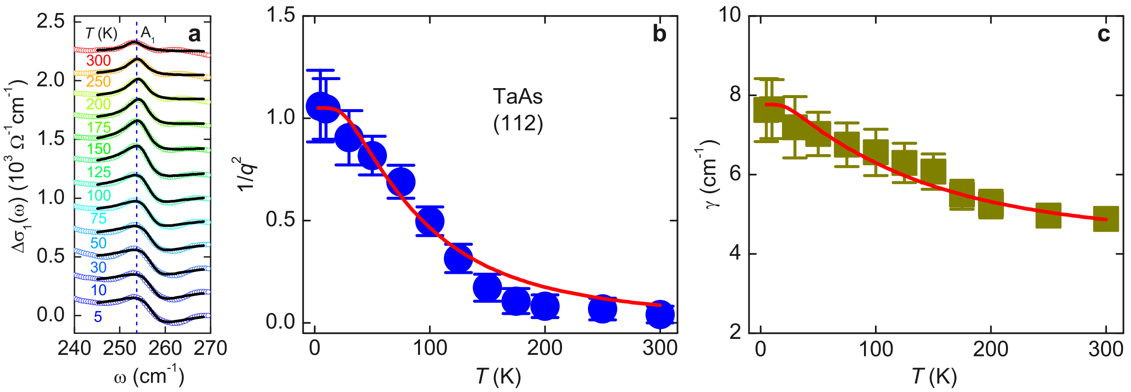

Supplementary Figure 4 | Fano fit and temperature dependence of fitting parameters. **a**, Line shape of the A_1 phonon, extracted from the optical conductivity measured at different temperatures on the (112) surface of TaAs. The black solid lines through the data represent the Fano fitting results. **b**, Temperature dependence of the Fano parameter $1/q^2$, which describes the asymmetry of the phonon line shape. The red solid curve is the least-square fit using Eq. (2) in the main text. **c**, The phonon linewidth γ as a function of temperature. The red solid curve denotes the least-square fitting result using the summation of Eq. (4) and Eq. (5) in the main text. Error bars in **b** and **c** are estimated by fitting the phonon line shape to the Fano equation in different frequency ranges at all measured temperatures.

Supplementary Note 1

Non-centrosymmetric TaAs crystallizes in the $I4_1md$ space group (No. 109)[1,2] with the irreducible vibrational representation $[A_1 + E] + [A_1 + 2B_1 + 3E]$[3], where the first term represents acoustic modes and the second term corresponds to optical modes. While all the optical modes are Raman active, only the A_1 and E modes are IR active. All of the optical phonons have been observed in a recent room-temperature Raman spectroscopy study[3]. The frequency ($\sim$253 cm^{-1}) of the phonon we observed in $R(\omega)$ agrees very well with the A_1 mode that was identified at $\sim$252 cm^{-1} by Raman spectroscopy. Furthermore, the E modes involve vibrations in the ab plane, while the A_1 mode arises from vibrations along the c axis[3]. The absence of the 253 cm^{-1} mode in $R(\omega)$ measured on the (001) surface, as shown in Supplementary Fig. 2, indicates that it is active along the c axis. These observations demonstrate that the 253 cm^{-1} phonon in $R(\omega)$ is associated with the A_1 mode.

Supplementary References

1. Weng, H., Fang, C., Fang, Z., Bernevig, B. A. & Dai, X. Weyl Semimetal Phase in Noncentrosymmetric Transition-Metal Monophosphides. *Phys. Rev. X* **5**, 011029 (2015).

2. Huang, S.-M. *et al.* A Weyl Fermion semimetal with surface Fermi arcs in the transition metal monopnictide TaAs class. *Nat. Commun.* **6**, 7373 (2015).

3. Liu, H. W. *et al.* Raman study of lattice dynamics in the Weyl semimetal TaAs. *Phys. Rev. B* **92**, 064302 (2015).
